# Efficient iterative Hi-C scaffolder based on N-best neighbors

**DOI:** 10.1186/s12859-021-04453-5

**Published:** 2021-11-27

**Authors:** Dengfeng Guan, Shane A. McCarthy, Zemin Ning, Guohua Wang, Yadong Wang, Richard Durbin

**Affiliations:** 1 Center for Bioinformatics, Harbin Institute of Technology, Harbin, 150001 China; 2 Department of Genetics, University of Cambridge, Cambridge, CB2 3EH UK; 3 Wellcome Sanger Institute, Wellcome Genome Campus, Cambridge, CB10 1SA UK; 4 Institute of Zoology, Chinese Academy of Sciences, Beijing, 100101 China

**Keywords:** Hi-C, Scaffolding

## Abstract

**Background:**

Efficient and effective genome scaffolding tools are still in high demand for generating reference-quality assemblies. While long read data itself is unlikely to create a chromosome-scale assembly for most eukaryotic species, the inexpensive Hi-C sequencing technology, capable of capturing the chromosomal profile of a genome, is now widely used to complete the task. However, the existing Hi-C based scaffolding tools either require a priori chromosome number as input, or lack the ability to build highly continuous scaffolds.

**Results:**

We design and develop a novel Hi-C based scaffolding tool, pin_hic, which takes advantage of contact information from Hi-C reads to construct a scaffolding graph iteratively based on N-best neighbors of contigs. Subsequent to scaffolding, it identifies potential misjoins and breaks them to keep the scaffolding accuracy. Through our tests on three long read based de novo assemblies from three different species, we demonstrate that pin_hic is more efficient than current standard state-of-art tools, and it can generate much more continuous scaffolds, while achieving a higher or comparable accuracy.

**Conclusions:**

Pin_hic is an efficient Hi-C based scaffolding tool, which can be useful for building chromosome-scale assemblies. As many sequencing projects have been launched in the recent years, we believe pin_hic has potential to be applied in these projects and makes a meaningful contribution.

**Supplementary Information:**

The online version contains supplementary material available at 10.1186/s12859-021-04453-5.

## Background

Genome assembly is still one of the most essential problems in bioinformatics. For eukaryotic genomes, which are typically hundreds to thousands of Mb long, the only way to obtain their sequences is through assembling from millions of much shorter fragments. Recent development of sequencing technologies, including next generation sequencing (NGS) and single molecule long read sequencing, has enabled thousands of novel species to be sequenced [1–4].

The inexpensive and high throughput NGS technologies boosted the rate of de novo genome sequencing. Numerous important species were sequenced by using NGS. For example, the first giant panda genome was sequenced and assembled using NGS data with various insert sizes by the SOAPDenovo [5] assembler in 2010 [6], which achieved a 40 kb contig N50 and an assembly size of 2.25 Gb, covering 94% of the real genome. However due to the complexity of the sequence and the limited read size, NGS assembly usually yields highly fragmented contigs, which impedes further genomic studies.

The advent of long read sequencing technologies, such as PacBio Single Molecule Real-Time (SMRT) sequencing technology and Oxford Nanopore Technologies (ONT), is now revolutionizing genome assembly studies. These technologies, which give read length two orders of magnitude or much longer than NGS, are leading to unprecedentedly complete and continuous genome assemblies. Chin et al. published a PacBio long read based de novo assembler “Falcon-unzip” for generating haplotype-phased assemblies [1] in 2016, where they described how the assembler could produce an Arabidopsis thaliana primary assembly with 8 Mb contig N50, which is about 8000 times larger than that of the SOAPDenovo assembly based on NGS reads.

Even though a long read based assembly itself can usually reach a megabase scale contig N50 [7], this is still far from routinely assembling to chromosome scale. Large genome assembly projects, such as Vertebrate Genomes Project (VGP), strategically combine multiple long range sequencing data, such as PacBio sequencing data, linked reads data [8], optical mapping data [9] and Hi-C data to construct reliably chromosome-scale assemblies. Hi-C in particular is empowered to build chromosome-scale assemblies [10, 11].

Hi-C sequencing technology is based on Chromosome Confirmation Capture technology, where fragments of DNA that are physically close are ligated with a labelled nucleotide at the ligation junction, enabling selective purification of chimeric DNA ligation junctions followed by deep sequencing [12, 13]. Since sequences on the same chromosome tend to be in proximity, Hi-C interactions provide informative evidence for ordering and orienting contigs, making use of two properties: (1) the intra-chromosomal interaction frequency is significantly higher than the inter-chromosomal interaction frequency; (2) the intra-chromosomal interaction frequency decays with genomic distance [10].

Several tools have been developed to achieve chromosome scale scaffolds based on Hi-C data in the last few years. DNATri [14] and LACHESIS [13] are perhaps the earliest tools using Hi-C reads for scaffolding. DNATri utilizes an average-linkage hierarchical clustering algorithm on a distance matrix derived from the contact matrix, to cluster and assemble the contigs. LACHESIS builds scaffolds through three steps: (1) Contig clustering: it tallies the number of links between contig pairs, and merges contigs using hierarchical agglomerative clustering until the cluster number is the same as a user-specified chromosome number; (2) contig ordering: it constructs a graph for each cluster, whose vertices are composed of the contigs and the edge weight is represented by the normalized link numbers. It then finds a “trunk” in the minimum spanning tree (MST) of this graph and adds vertices not belonging to the “trunk” back to it; (3) contig orienting: it builds a weighted directed acyclic graph (WDAG) for each direction of a contig, and determines the contig orientations by seeking the maximum weighted path in the WDAG. 3D-DNA [11], SALSA1 [15] and SALSA2 [16] are three more recent tools. All these tools correct the input draft assembly before scaffolding. 3D-DNA applies the best neighbor strategy to assemble the contigs into one “megascaffold” and then breaks it into a number of chromosomes. SALSA1 collects the link numbers between contig pairs and normalizes them by a count of enzyme cutting sites; it then orders and orients the contigs through building a directed acyclic graph (DAG) by processing the maximum weighted links iteratively. SALSA2 is an upgrade from SALSA1 that can process an input assembly graph. SALSA2 also uses the best neighbor strategy to select the best joining candidates and reduce inversion errors, and it can stop scaffolding iteration automatically.

Here, we present another Hi-C scaffolding approach and provide an implementation in the pin_hic software. Pin_hic uses N-best neighbor strategy to order and orient contigs and an iterative weighted linking approach to further elongate them, which we show can obtain chromosome-scale scaffolds without requiring to know the chromosome number. It also applies a robust method to diagnose misjoins regardless of scaffold length, which improves the scaffolding accuracy. Furthermore, to maintain the information of the scaffolding graph, we also proposed a new format “SAT”. In our experiments on three draft assemblies from three different species, pin_hic outperforms the state-of-art tool SALSA2 by up to 1.4 times in continuity while achieving higher or comparable accuracy, and up to 1.7 times in speed.

## Implementation

### Overview

Given raw Hi-C reads, a draft assembly and a number of iterations, we generate chromosome-scale scaffolds with the following steps:

- Step 1. Map the Hi-C read pairs to the draft assembly independently.
- Step 2. Build a contact matrix counting the number of linking read pairs between contig ends based on the bam files, and the SAT file if it exists.
- Step 3. Construct a scaffolding graph based on N-best neighbors of contigs with the contact matrix, make joins, and output the results in “SAT” format.
- Step 4. Check if all iterations are finished, if so, go to Step 5, otherwise repeat Step 2 to 4 using the alignment files and the new SAT file output in the last step as inputs.
- Step 5. Search for misjoins in the final SAT file, break at any detected mis-joins, and output the final scaffold sequences.

The whole pipeline is illustrated in Additional file 1: Fig. S1. Major steps are described in details in the following sections.

### Contact matrix calculation

Given raw Hi-C reads and a draft assembly, we first map the reads to the draft assembly with “bwa mem” using settings of “-SP” to skip read pairing and mate pair rescue [17], and a relatively large mismatch penalty 10 to seek more consistent target sequences.

Given the resulting alignment bam files and the current contigs (scaffolds in the next iteration), we then calculate the contact matrix with the following steps:

- Step 1. Each contig $i$ is split into three equal parts, the middle part is ignored in calculation, and the first and third part denoted as contig $ih$ and contig $it$ represents 5’ and 3’ ends of the contig respectively.
- Step 2. A list of tuples $t$ $(jy,c_{ix,jy})$ is initialised for each contig ix to record the number of linking read pairs (contact number) with contig $jy$, while $jy$ is the contig index and $c_{ix,jy}$ is the contact number.
- Step 3. All the read pairs in the bam files are evaluated, with a read pair contributing to the counts only if it satisfies the following three conditions:
  - Both 5′ ends of a read pair are mapped to different contigs unambiguously without soft or hard clippings.
  - Mapping qualities are no less than $q$ (default: 10).
  - Not a duplicated read pair.
- Step 4. All qualified read paris are tallied for contig $ix$ and contig $jy$, and the tuples are output as a contact matrix.

Here, pin_hic splits contigs into three equal parts, to reduce misjoins. This is different from other tools like SALSA2, LACHESIS, which split contigs into two halves and accumulate their arrangement evidence. Since in a regular case, more contacts will occur at the ends of the contigs, the unexpected contacts occurring in the middle of the contigs can lead to misjoins. Besides, unlike SALSA2 which generates an intermediate bed file during each iteration, pin_hic always uses the original bam file(s) as inputs which allows it to use much less disk space.

Normally, to produce more continuous scaffolds, users may need to perform this contact matrix calculation multiple times, they may expect to know the scaffolding graphs in each iteration as well. In such a case, pin_hic uses its own SAT format to record the scaffolding graph. Details of the SAT format, which is derived from the widely used “graph fragment assembly” (GFA) format [18] and was designed to carry more details of the graph than the traditional “AGP” format, are described in the “SAT format” section. In each iteration, if the SAT file is used as an input, the paths (scaffolds) will be construct first and each original contig in the draft assembly will keep a record of its corresponding scaffold and their start positions on that scaffold, so that target positions in the bam files can be converted into positions in the scaffolds internally by pin_hic.

### Contact matrix normalization

After we obtain the contact matrix $M$, we normalize it with Eq. 1 to remove the effects of noisy hits in long contigs.

1 $$nc= \frac{{c}_{ix,jy}}{{l}_{i}/3+{l}_{j}/3}$$

where $l_{i}$ and $l_{j}$ are the length of contig $i$ and contig $j$ respectively. We also ignore contig pairs with contact number less than $w$ to avoid short contigs having fairly large normalized contact numbers. For a contig $ix$, we choose contig $jy$ with top $N$ normalized contact numbers as its N-best neighbors. Here, we use summation of contig lengths to reduce noises from long contigs, some other methods like LACHESIS also use multiplication of contig lengths as the denominator in Eq. 1 for normalization. Based on our experiments, we showed that the former is more useful to decrease misjoins.

### Scaffolding graph construction

**Fig. 1:**
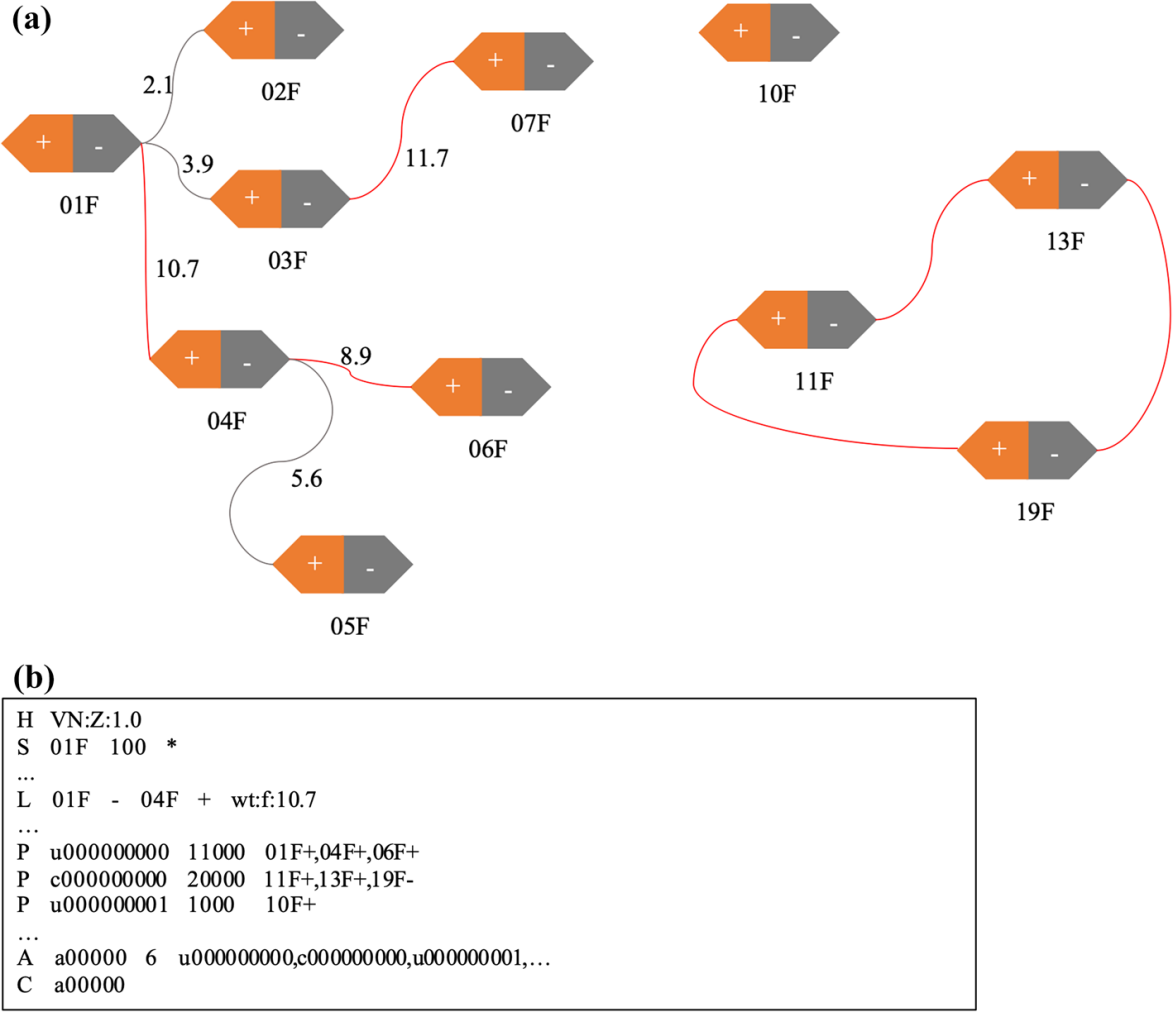
Scaffolding graph and SAT format example. **a** A scaffolding graph. The graph containing 22 vertices and 9 edges is formed by 11 contigs, each contig is split into two vertices, the text below each hexagon is the contig name. Numbers along with the edges are normalized weights, and the grey edges are removed by the pruning process. **b** A SAT example. All the contigs are represented as sequences (‘S’) in the SAT file, edges are defined as links and tagged as ‘L’, three scaffolds obtained from **a** are labelled as paths (‘P’) and three scaffolds are gathered in the assembly set tagged as ‘A’, and current assembly is tagged as ‘C’

After normalization, we then construct a scaffolding graph through our N-best neighbor algorithm. The time complexity of the algorithm is $O(|V | + |E|)$, where $|V |$ represents the number of vertices, $|E|$ is the number of edges in the graph. The steps of the algorithm (Algorithm 1) are described as followed:

- Step 1. Define $SG = (V, E)$ as an undirected graph, whose vertices are composed of the 5′ and 3′ ends of the contigs mentioned in the last section (Fig. 1a): one contig $i$ generates two vertices $V_{ih}$ and $V_{it}$, where $V_{ih}$ represents the 5′ of the contig and $V_{it}$ the 3′.
- Step 2. Create edges $E$ based on the matrix $M$. An edge exists between $V_{ia}$ and $V_{jb}$, if and only if $V_{i}$ is among the N-best neighbors (default: 3) of $V_{j}$ and vice versa.
- Step 3. Perform a pruning process so that there is at most one edge per vertex, by only keeping the edge with unique maximum weight. If two or more edges have the same maximum weight, all edges linked to the vertex are removed.
- Step 4. Add edges between the 5’ and 3’ vertex of each contig, and traverse the graph to find all the components, which are scaffolds. If there is a loop in the scaffolding graph, we choose an arbitrary vertex as the beginning and its predecessor as the end. At last, we output all the vertices, edges, paths and other information about the scaffolding graph, such as the weight between a contig pair, into a SAT file.

Figure 1 gives an example in which there are 22 vertices and 9 edges in the graph. After pruning, all the grey edges are deleted and only five red edges remain. There are 6 paths in the graph, 3 of them formed by a single contig, 1 cyclic path is built by contig 11F, 13F and 19F, two acyclic paths are formed by contig 01F, 04F, 06F and 03F, 07F respectively.
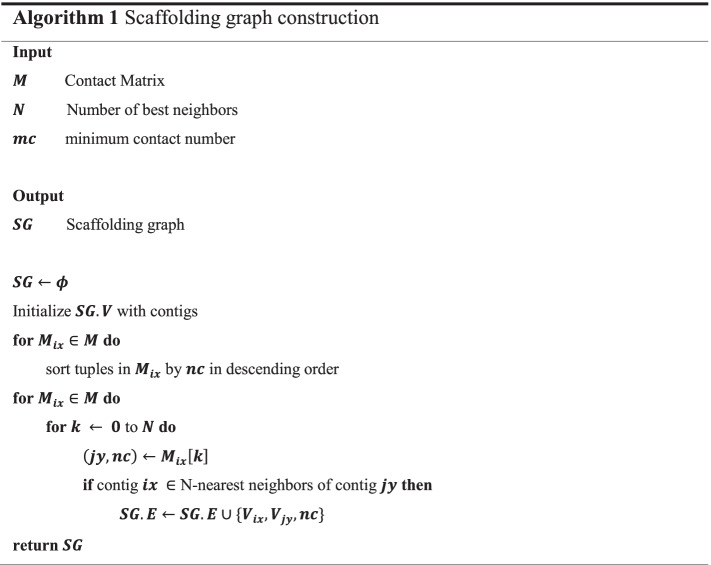


### Misjoin detection

Even though we normalize edge weights by contig lengths, a large scaffold, especially a complete chromosome, based on our observations, is prone to be misjoined with other chromosomes (Fig. 4a). To resolve this problem, we employ an extra step after scaffolding to make breaks at the potential misjoins.

During the procedure, we take the bam files and the final scaffolding SAT file as inputs, and obtain scaffolds and their corresponding contigs from the SAT file. We gather read pairs which meet the following requirements:

- Both 5' ends of a read pair are mapped to the same contig or adjacent contigs in the same scaffold unambiguously without soft or hard clippings.
- Mapping qualities are no less than q (default: 10).
- Not a duplicated read pair.

We then use these read pairs to calculate the physical spanning coverage for each base, and collect maximum coverage for each contig and each join between adjacent contigs. We then break at the joins whose maximum coverage is less than p% (default 30%) of that of each of their neighboring contigs. This method is based on the assumption that misjoins between contigs tend to have many fewer spanning read pairs than equivalent regions within the contigs themselves.

### SAT format

To retain the scaffolding graph information and employ the information in further process, we define a new file format called “SAT” format, which is inspired by the “GFA” format and extended to keep scaffolding information.

We use the “S” tag to represent the contigs with the second field after “S” being the contig ID, the third field the contig length, and forth optional field for contig sequence.

We use “L” to represent edges, with a plus or minus sign to represent the 5' and 3' ends of the contig respectively, and we also add a tag “wt” to record the edge weight.

We use “P” to represent a path (scaffold), with the second field of “P” being the scaffold ID, the third the scaffold length, and the fourth being the components of the scaffold.

Then we add further tags “A” to represent the set of scaffolds and “C” to represent the current scaffold set. These tags support the iterative usage of the file.

Figure 1 gives a simple example of SAT format. To facilitate our users, we have supplied a tool called “satool” (https://github.com/dfguan/satool) to visualize and convert SAT format to the AGP format used by NCBI and others to represent chromosomal scaffolds.

## Results

To assess the performance of pin_hic, we conducted three experiments on three different VGP assemblies, and compared our results with the state-of-art scaffolding tool SALSA2 and 3D-DNA. The benchmarks include computational resource consumption, scaffolds continuity and correctness, since 3D-DNA implements parallelization internally, we ignored its consumption of computational resource.

Both SALSA2 and pin_hic were run in default mode on a LSF HPC cluster. SALSA2 read alignment and filtering were done by the recommended Arima mapping pipeline, pin_hic used the alignments directly from bwa and filtered the read pairs internally. The CPU runtime and peak memory for scaffolding are collected by the LSF platform.

### Assembly collection

We collected three VGP assemblies, which were all built at chromosome scale, including an *Anabas testudineus* (common name: climbing fish, At for short) assembly, a *Takifugu rubripes* (common name: Tiger puffer, Tr for short), and a *Calypte anna* (common name: Anna’s hummingbird, Ca for short). All assemblies were split at each run of Ns to get the original contigs. Assembly metrics for each species is listed in Table 1.

**Table 1:** The statistic of genome assembly metrics

	NCBI Assembly ID	Genome size (Mb)	# Scaffold	Scaffold N50 (Mb)	# Contig	Contig N50 (Mb)	BUSCO complete genes (%)
At	GCA_900324465.2	556	50	25.06	316	7.06	97.5
Tr	GCF_901000725.2	384	128	16.71	530	3.14	96.6
Ca	GCA_003957555.2	1060	159	74.08	586	14.52	93.9

For At, we used the fAnaTes1.2 assembly with a scaffold N50 of 25.06 Mb, containing 316 contigs with 7.06 Mb contig N50. The Tr assembly has a scaffold N50 of 16.71 Mb, having 530 contigs with 3.14 Mb contig N50; Ca assembly has a scaffold N50 of 74.08 Mb, after removing two contigs less than 1 kb, it has 586 contigs and contig N50 of 14.52 Mb.

The split At, Tr and Ca assemblies have 97.5%, 96.6% and 93.9% complete genes count using BUSCO [19] analysis, showing all the contigs having a high completeness. Below we use Ats to refer to the split At assembly, Trs for the split Tr assembly, and Cas for the split Ca assembly.

### Hi-C reads collection and preprocessing

As is displayed in Table 2, we collected 341 M, 128 M and 515 M Hi-C read pairs for At, Tr and Ca respectively, which were all sequenced in Arima Genomics, using MboI and HinfI enzyme. Before scaffolding, we mapped all Hi-C read pairs to the contigs, and preprocessed the Hi-C reads with recommended Arima mapping pipeline (https://github.com/ArimaGenomics/mapping_pipeline) for SALSA2.

**Table 2:** The statistic of Hi-C reads alignment results

	# Read pairs (M)	# Intra-contig read pairs (M)	# Inter-contig read pairs (M)
Ats	341	155	85
Trs	128	52	32
Cas	515	193	198

After mapping, we performed a statistic on read pairs aligned intra and inter contigs. As is shown in Table 2, all split assemblies have over 25% mapped to inter contigs, which can be used for scaffolding. Ca has most inter-contig read pairs, although Tr has only 128 M Hi-C read pairs, it still has 32 M inter-contig read pairs for scaffolding.

### Scaffolding results evaluation

The scaffolding results are shown in Table 3, in which the best results are highlighted in bold. In the experiments, both SALSA2 and 3D-DNA were run in default settings without error correction before scaffolding. Pin_hic was run in default settings which uses three iterations, summation normalization and three-part split method. We assessed their accuracy using QUAST-LG [20] with the chromosome-scale assemblies mentioned in the last section.

**Table 3:** The scaffolding results of primary contigs from three species

	# sequence	Largest (Mb)	NG50 (Mb)	NGA50 (Mb)	Reloc	Inv	Tran	RAM (GB)	Runtime (CPU hrs)
Ats-org^1^	316	16.97	7.06	–	–	–	–	–	–
Ats-phc^2^	93	34.17	24.11	17.83	67	**10**	**0**	2.94	**1.62**
Ats-sal^3^	124	28.24	20.93	12.47	**64**	18	**0**	**2.22**	1.80
Ats-3d^4^	95	**232.83**	**131.30**	**18.81**	85	19	39	–	–
Trs-org	530	13.38	3.28	–	–	–	–	–	–
Trs-phc	267	28.10	15.75	**9.32**	**62**	**13**	**9**	**0.98**	**0.78**
Trs-sal	273	26.82	11.11	5.85	82	16	10	1.44	1.08
Trs-3d	653	**28.63**	**16.81**	6.00	286	35	108	–	–
Cas-org	586	48.75	14.52	–	–	–	–	–	–
Cas-phc	242	**184.75**	44.74	28.65	106	**9**	12	**4.54**	**2.43**
Cas-sal	299	179.32	35.40	22.55	**85**	12	**6**	4.74	4.02
Cas-3d	14	84.57	**84.57**	**35.40**	196	24	113	–	–

The best results are highlighted in bold

^1^Original primary contigs

^2^pin_hic scaffolding result

^3^SALSA2 scaffolding result

^4^3D-DNA scaffolding result

#### Memory consumption and speed

As is shown in Table 3, pin_hic is more efficient than SALSA2, its speed is around 11%–65% faster than that of SALSA2. And it consumes less memory for all the cases except Ats scaffolding, in which it still has a comparable memory consumption.

#### Scaffold continuity

As is shown in Table 3, 3D-DNA owns the largest scaffolds and NG50s for all the species, except for Cas where pin_hic is the winner of the largest scaffold, indicating its capacity of generating more continuous than the other tools. However, its largest scaffold of Ats is about 7 times larger than that of the reference assembly, which implies misjoins in the scaffold. By contrast, both pin_hic and SALSA2 have obtained the largest scaffolds with proper lengths, especially for pin_hic, the largest 34.17 Mb scaffold is consistent with the VGP curated assembly, which shows its ability in producing correct scaffolds. For pin_hic, all NG50s are improved by at least 3 times than those of the original assemblies, the scaffold NG50 of Trs is improved by 4.8 times.

NGA50, reflecting both scaffolding correctness and continuity, is another important measurement for scaffold evaluation. Both 3D-DNA and pin_hic have larger NGA50s than that of SALSA2s. However, 3D-DNA achieves a higher NGA50 with a cost of scaffolding accuracy, which we will discuss in the following section. The NGA50s of pin_hic are at least 27% larger than those of SALSA2, and 59% larger than SALSA2 on Trs scaffolds, which indicates pin_hic can generate highly accurate assembly.

#### Scaffold correctness

As is shown in Table 3, pin_hic has less misjoins than SALSA2 on Ats and Trs scaffolds, and has 24 more misjoins than SALSA2 on Cas scaffolds. Through analyzing standard deviation (SD) on Cas contig lengths, we discovered the misjoin number may be proportional to SD, a larger SD is prone to lead to more misjoins in pin_hic scaffolds. Both SALSA2 and pin_hic has majority of relocation errors, taking at least 74% of all their misjoins. 3D-DNA has most misjoins, especially it contains much more translocation error than the other tools, which needs further manual correction as it is designed.

**Fig. 2:**
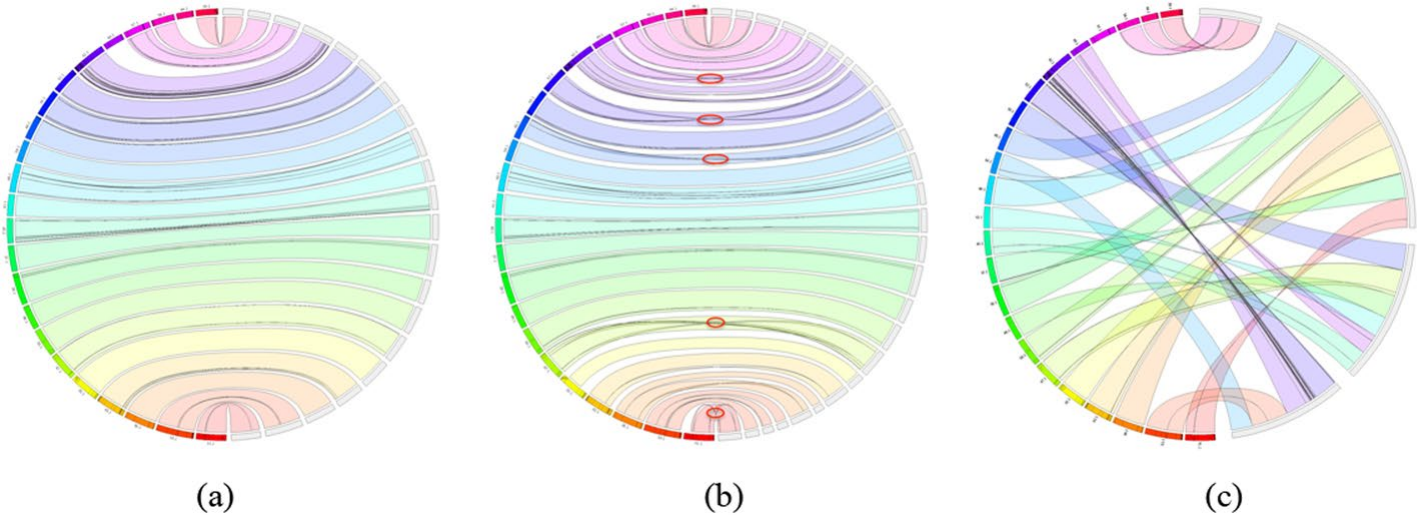
Scaffolds consistency plots of Ats scaffolds. **a** Pin_hic scaffolds, **b** SALSA2 scaffolds and **c** 3D-DNA scaffolds. The largest 21, 27 and 4 scaffolds from pin_hic, SALSA2 and 3D-DNA scaffolds, consisting of 90% (NG90) of the genome, are aligned to the VGP fAnaTes1.2 assembly. The chromosomes are displayed incrementally from 1 to 23 on the left side and the scaffolds are located on the right side of the ring. Connections show aligned regions over 10 kb. Large-scale mis-assemblies are visible as interrupting ribbons. Pin_hic scaffoldings are neat, no clear misjoins are found, while SALSA2 scaffolds contain about 5 relocations, and 3D-DNA scaffolds contain numerous misjoins

To further validate the correctness of the scaffolds, we made circos plots to display the consistency between the scaffolds and the reference genomes by using Jupiterplot [21]. The plots of Ats scaffolds are shown in Fig. 2. In these figures, scaffolds consisting of 90% of the reference genome size, are selected to map to the VGP fAnaTes1.2 assembly, the chromosomes of the assembly are displayed on the left side and the scaffolds on the right, the interrupting ribbons are the visible mis-assemblies. 3D-DNA contains numbers of misjoins which requires further manual curation. As for SALSA2, although no translocation occurs, there are at least 5 visible relocations within its scaffolds, while no apparent mis-assemblies are observed in pin_hic scaffolds, which implies its higher accuracy than SALSA2 in the Ats scaffolds. Additional file 1: Figs. S4, S5 showing Cas and Trs circos plots indicating the similar situations.

### Misjoin detection evaluation

**Fig. 3:**
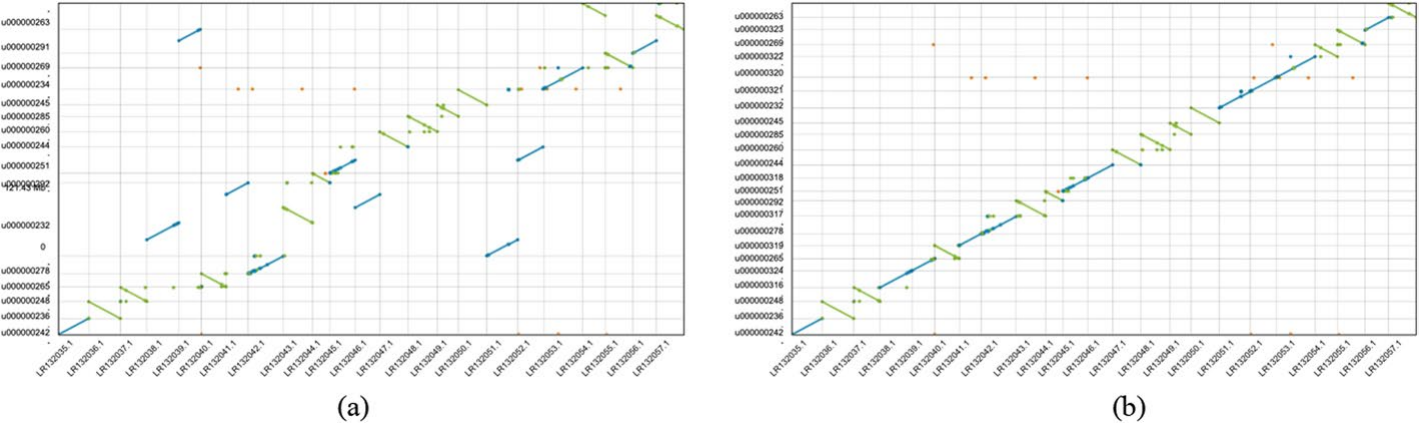
Alignment dotplots for pin_hic Ats scaffolds before and after mis-join detection. **a** pin_hic scaffolds alignment before misjoin detection and **b** pin_hic scaffolds alignment after misjoin detection. Both scaffolds are aligned to the VGP fAnaTes1.2 reference genome. Before misjoin detection, several chromosome-scale scaffolds are concatenated together, after the process, all the large misjoined scaffolds are corrected, the scaffolds are aligned to the reference genome more consistently

The final step in pin_hic scaffolding process is misjoin detection, to prove efficacy of the method, we mapped the scaffolds onto their reference genomes, and made the dotplots based on the alignment results by a public Dotplot tool (https://github.com/dnanexus/dot). As is demonstrated in Fig. 3, where the Ats scaffolds were mapped to its reference genome. Before misjoin detection, it contains several misjoins, a few independent chromosomes are chained together. For example, the scaffold “u000000232” is made up of five complete chromosomes. After misjoin detection, all the visible misjoined contigs are separated, the scaffolds is aligned consistently to the reference, which proves robustness of the misjoin detection method. We observed same situations for Trs and Cas scaffolds in Additional file 1: Figs. S2, S3.

**Fig. 4:**
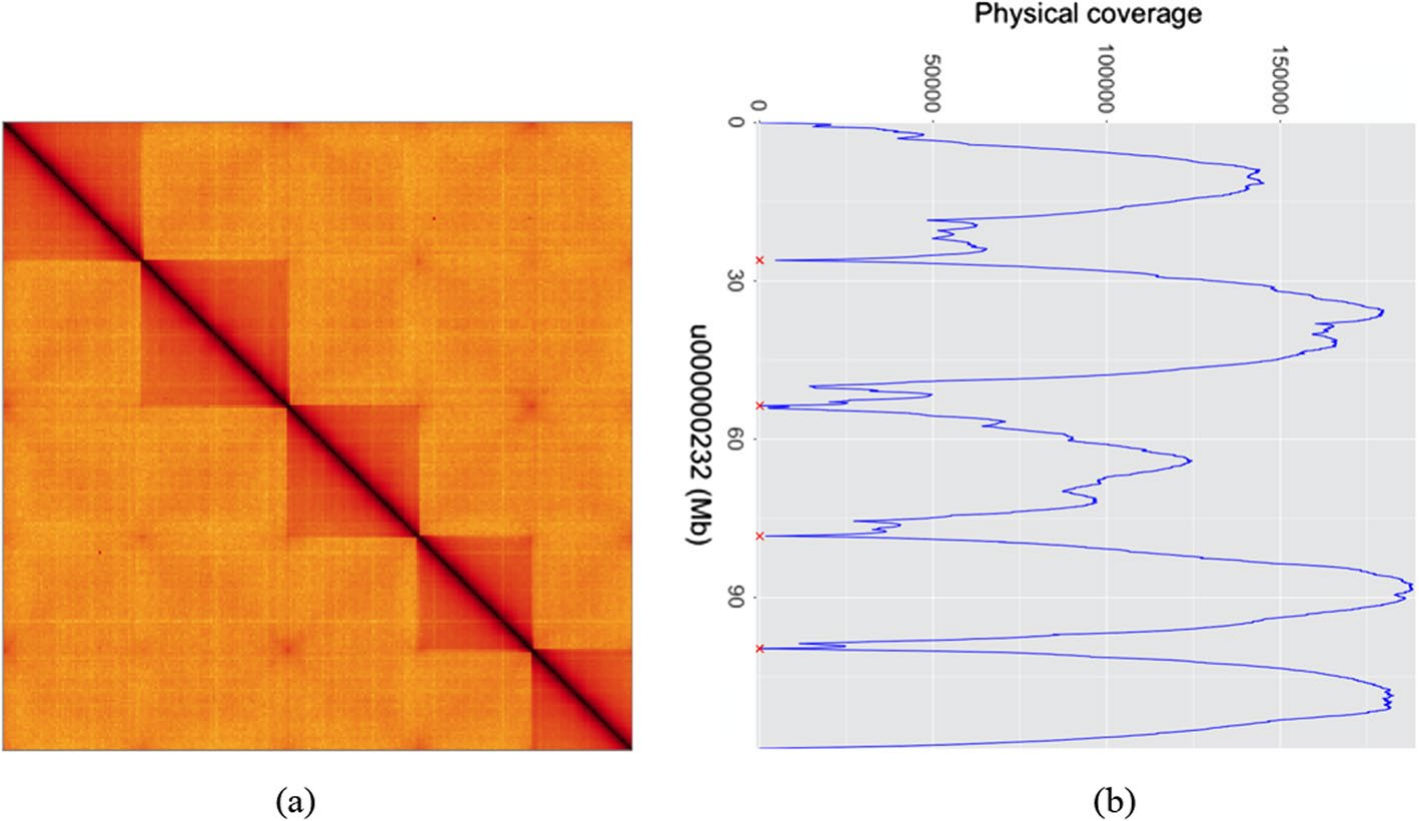
Misjoin detection on a Ats scaffold. **a** HiGlass heatmap of “u000000232” in Ats scaffolds. The five independent blocks showing no evidence to be joined together were put together due to noises. **b** Physical coverage plot based on the misjoin detection algorithm. The joins which have much lower coverage than their neighbors are recognized as misjoins and broken (the red crosses)

In Fig. 4, we give an example to explain the details of the misjoin identification method. We extracted the scaffold “u000000232” from the Ats scaffolds before misjoin detection, which is composed of 43 contigs and 121 Mb long, and made a heatmap using HiGlass [22] based on the read pairs map to this scaffold and a coverage plot. As is illustrated in Fig. 4, the HiGlass heatmap implies five independent chromosomes were joined together, meanwhile the physical coverage plot shows fairly low local minima at the misjoins. The program made 4 breaks (red crosses in the coverage plot) in the scaffolds, and separated the scaffold into six scaffolds correctly, which proves the method can work properly to find the misjoins. The other low-coverage joins caused by missing or short sequences are not broken.

### Pin_hic performance in different modes

To measure the effects of different normalization and split methods. Pin_hic were implemented and tested in four different modes: default, 3m, 2s, 2m. Default mode represents the default settings of pin_hic (i.e., three-part split and summation normalization method). The number before the letter represents how many parts a contig is split into, and m, s represents multiplication and summation normalization method respectively. All tests were run in three iterations. The results are shown in Table 4, best results are highlighted in bold.

**Table 4:** The scaffolding results in different modes

	# sequence	Largest (Mb)	NG50 (Mb)	NGA50 (Mb)	Reloc	Inv	Tran	RAM (GB)	Runtime (CPU hrs)
Ats-phc^1^	93	**34.17**	**24.11**	17.83	**67**	**10**	**0**	2.94	**1.62**
Ats-phc-2s	89	32.64	**24.11**	18.39	72	12	**0**	3.76	1.72
Ats-phc-3m	**53**	28.84	21.98	**18.81**	73	18	5	2.91	1.84
Ats-phc-2m	**53**	28.34	21.83	**18.81**	71	15	5	2.91	1.73
Trs-phc	267	28.10	**15.75**	9.32	**62**	**13**	**9**	**0.98**	0.78
Trs-phc-2s	250	28.04	15.45	9.32	70	21	**9**	0.99	0.63
Trs-phc-3m	207	27.95	14.73	8.94	81	16	23	0.82	**0.50**
Trs-phc-2m	**191**	**29.07**	14.25	**9.52**	81	26	12	**0.98**	0.63
Cas-phc	242	**184.75**	44.74	**28.65**	**106**	**9**	**12**	**4.54**	**2.43**
Cas-phc-2s	223	326.93*	43.88	**28.65**	111	12	18	**4.54**	2.88
Cas-phc-3m	163	170.93	**45.83**	**28.65**	119	11	32	**4.54**	2.62
Cas-phc-2m	**154**	107.91	39.24	**28.65**	115	14	32	**4.54**	3.11

The best results are highlighted in bold

*Chromosomes are misjoined

^1^pin_hic default scaffolding mode

As is shown in the table, different mode of pin_hic has slight effects on resource consumption and shows no clear pattern.

As for scaffolding continuity, pin_hic 2m mode has the largest NGA50 than the other modes, and has the least scaffold numbers, however its NG50 and largest scaffold is smaller than the default mode.

As for scaffolding correctness, we observed more misjoins, especially more translocation errors, in 3m and 2m mode. To balance scaffolding efficiency, scaffolding correctness and continuity, pin_hic use three-part split and summation normalization method as its default mode.

### Pin_hic performance with multiple iterations

**Fig. 5:**
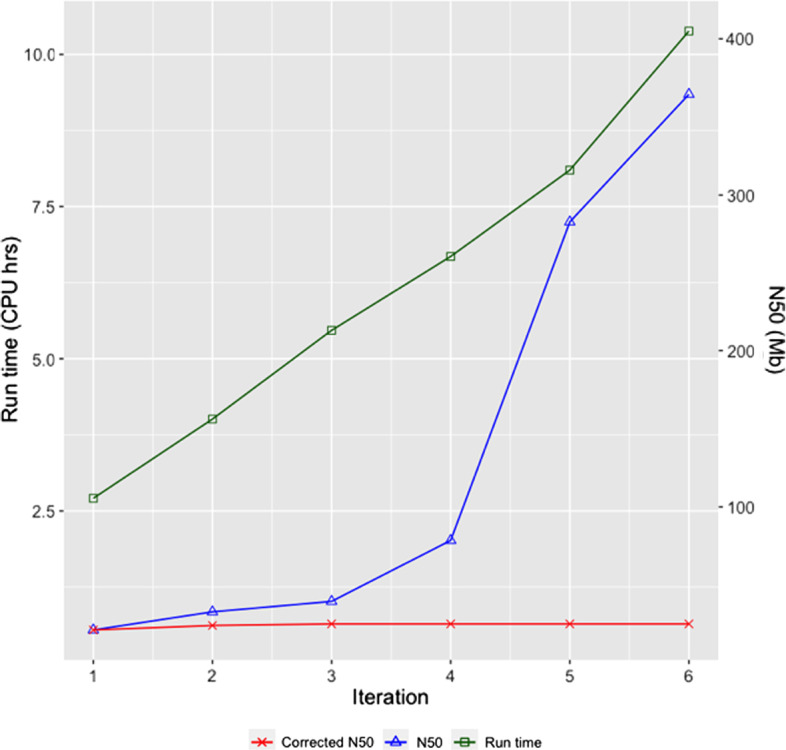
pin_hic Ats scaffolding results of multiple iterations. The runtime (dark green line) increases linearly with the iterations, while the scaffold N50 grows rapidly at the second and third round, it finally reaches 364 Mb at round 6. Even though the contigs are highly expanded, the corrected scaffold N50 keeps constant at 25 Mb since the third round

To investigate the effects of number of iterations on pin_hic performance, we ran pin_hic from 1 to 6 iterations on all the split assemblies, and collected N50s, corrected N50s after misjoin detection and CPU runtime for each iteration. Figure 5 shows the performance for Ats scaffolds. As is displayed, the CPU runtime (the blue line) is almost linear to the iteration numbers. Scaffold N50 (the green line) before misjoin detection increases drastically from 21.23 Mb (1st iteration) to 39.55 Mb (3rd iteration), which indicates most true positive links are exhausted during these periods. It finally reaches the scaffold N50 of 364.44 Mb. This large scaffold N50 is not affecting the misjoin method, the corrected N50 remains constant at 25.18 Mb scaffold N50 after the third round, which reflects robustness of the mis-join detection algorithm. Pin_hic iteration performances for the other two scaffolds are demonstrated in Additional file 1: Fig. S6.

## Conclusions

Building an accurate and chromosome-scale assembly is still one of the major challenges in genomic studies. In this paper, we proposed a new Hi-C scaffolding method for generating chromosome-scale scaffolds through iterative weighted linking, it uses N-best neighbor strategy to resolve non-reciprocal best neighbor issue and exploit all possible links, and a robust method to discover misjoins in scaffolds and improve scaffolding accuracy based on comparison of maximum physical coverages of the joins and their neighboring contigs, which is theoretically and practically unaffected by the scaffold lengths. Moreover, we defined a novel “SAT” format to keep a scaffolding graph, which can be used in further genomic analysis, such as manual curation.

Through our experiments on four long-read based de novo assemblies from three different species, we demonstrate that pin_hic can generate more continuous assembly than the start-of-art tool SALSA2, while achieving higher or comparable accuracy than 3D-DNA and SALSA2, it is also proved to be more efficient. Further, pin_hic program is implemented in C programming language, its only dependency is zlib, which makes it easy to compile and install, and easy to be integrated into modern assembly pipelines.

Although pin_hic has great potential to be applied in the real de novo sequencing projects, it required some further improvements: (1) It assumes the input assembly contains no mis-assemblies, so no module is designed to resolve chimeric assemblies, the misjoin detection algorithm can make breaks on joins, however it can not find mis-assemblies within a contig; (2) It still depends on users to specify the number of iterations, however users may set up a small number leading to short scaffolds. Although misjoin detection method is not affected by the scaffold lengths, user can choose a large number to produce more continuous results, it is useful to develop a automatic stop mechanism to save runtime. (3) Unlike mate pair reads, the distance and the relative orientation between Hi-C read pairs are not known, which makes it hard to determine gap sizes in a scaffold.

All the assemblies used in this study are based on long reads, which have natural advantages in read length over NGS reads and are able to span moderately repetitive regions and allow those regions to be built correctly. The advances in long reads assemblers have been stimulating the prosperity of long reads assembly. With long reads, we are capable of generating more continuous, more correct and more complete genomes than ever. This has enabled us to produce more accurate structural variant calling results, discover more novel genes, etc. However due to the complexity of eukaryotic genomes, long reads assemblies are typically inadequate to provide complete profile of the genomes, one still needs long-range sequencing technologies such as Hi-C to restore chromosomal structures of the assemblies.

The advent of long read and long-range Hi-C sequencing technology has opened a new era, in which the chromosome-scale assemblies can be generated automatically and productively. As many de novo sequencing projects have been launched in recent years, such as Darwin Tree of Life (DToL) Project (https://www.sanger.ac.uk/ science/collaboration/darwin-tree-life-project) and Vertebrate Genomes Project (VGP) (https://vertebrategenomesproject.org), we believe pin_hic has potential to be applied in these projects and accelerates production of chromosome-scale assemblies in the near future.

## Availability and requirements

- Project name: pin_hic.
- Project home page: https://github.com/dfguan/pin_hic.
- Operating system(s): Linux, MacOS.
- Programming language: C.
- Other requirements: gcc.
- License MIT Any restrictions to use by non-academics: None.

## Supplementary Information

**Additional file 1.** Supplementary note.

## Data Availability

Datasets used in the experiments are listed as follows: At: The original assembly and reference genome is available at NCBI with Genbank accession: GCA 900324465.2. The Hi-C data is deposited in NCBI with accessions ERR4179331-ERR4179339 and can also be downloaded with the command: “aws s3 -no-sign-request sync s3://genomeark/species/Anabas_testudineus/fAnaTes1/genomic_data/arima/”. Ca: The original assembly and reference genome is available at NCBI with Genbank accession: GCF 003957555.1. The Hi-C data can be downloaded with the command: “aws s3 -no-sign-request sync s3://genomeark/species/Calypte_anna/bCalAnn1/genomic_data/arima/”. Tr: The original assembly and reference genome is available at NCBI with RefSeq accession: GCF 901000725.2. The Hi-C data is deposited in NCBI with accessions ERR4179374-ERR4179378, and can also be downloaded with the command: “aws s3 -no-sign-request sync s3://genomeark/species/Takifugu_rubripes/fTakRub1/genomic_data/arima/”.
