# Correction to: Efficient iterative Hi-C scaffolder based on N-best neighbors

**DOI:** 10.1186/s12859-021-04537-2

**Published:** 2021-12-31

**Authors:** Dengfeng Guan, Shane A. McCarthy, Zemin Ning, Guohua Wang, Yadong Wang, Richard Durbin

**Affiliations:** 1 Center for Bioinformatics, Harbin Institute of Technology, Harbin, 150001 China; 2 Department of Genetics, University of Cambridge, Cambridge, CB2 3EH UK; 3 Wellcome Sanger Institute, Wellcome Genome Campus, Cambridge, CB10 1SA UK; 4 Institute of Zoology, Chinese Academy of Sciences, Beijing, 100101 China

## Correction to: Guan et al. BMC Bioinformatics (2021) 22:569 10.1186/s12859-021-04453-5

Following publication of the original article [1], the authors identified an error in Fig. 1. The correct figure is given below.

**Fig. 1:**
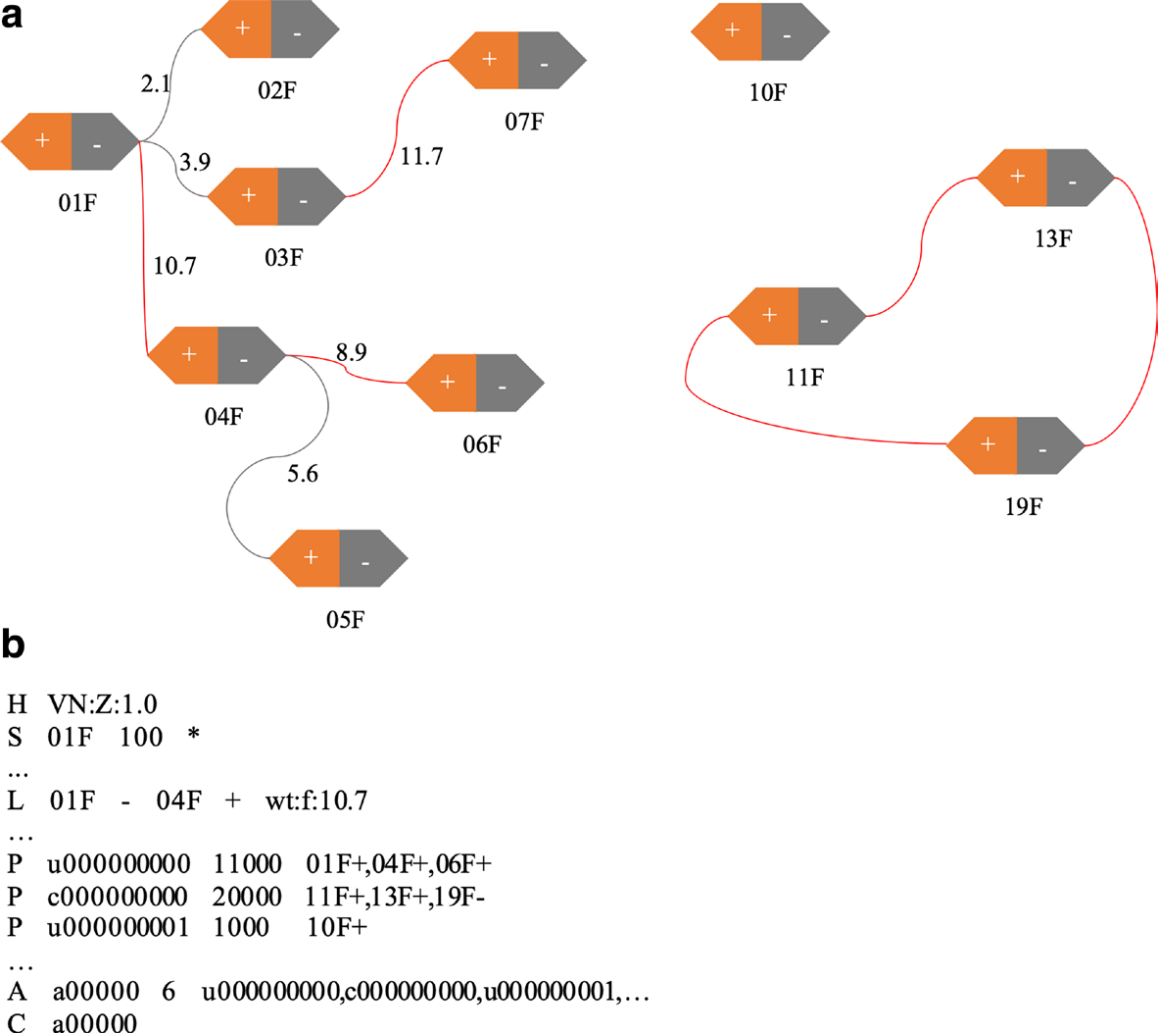
Scaffolding graph and SAT format example. **a** A scaffolding graph. The graph containing 22 vertices and 9 edges is formed by 11 contigs, each contig is split into two vertices, the text below each hexagon is the contig name. Numbers along with the edges are normalized weights, and the grey edges are removed by the pruning process. **b** A SAT example. All the contigs are represented as sequences (‘S’) in the SAT file, edges are defined as links and tagged as ‘L’, three scaffolds obtained from a are labelled as paths (‘P’) and three scaffolds are gathered in the assembly set tagged as ‘A’, and current assembly is tagged as ‘C’

The original article [1] has been corrected.
