# Supplementary material for: Efficient iterative Hi-C scaffolder based on N-best neighbors
Source: BMC Bioinformatics. 2021 Nov 27;22:569. doi: 10.1186/s12859-021-04453-5 (PMC8627104; doi:10.1186/s12859-021-04453-5)
Supplement: Supplementary file 1 — Additional file 1. Supplementary note. [file 12859_2021_4453_MOESM1_ESM.pdf]

# Pin_hic supplementary note

Dengfeng Guan[1,2,4], Shane A. McCarthy[2,3], Zemin Ning[3], Yadong Wang[1], Guohua Wang[1], and Richard Durbin[2,3]

[1]Center for Bioinformatics, Harbin Institute of Technology, Harbin, 150001, China
[2]Department of Genetics, University of Cambridge, Cambridge, CB2 3EH, UK
[3]Wellcome Sanger Institute, Wellcome Genome Campus, Cambridge, CB10 1SA, UK
[4]Institute of Zoology, Chinese Academy of Sciences, Beijing, 100101, China

## Contents

**1 Software tools** **2**

**2 Scaffolding commands** **2**
2.0.1 Pin_hic command lines . . . 2
2.0.2 SALSA2 command lines . . . 2
2.0.3 3D-DNA command lines . . . 2

**3 Supplementary tables and figures** **2**

# 1 Software tools

The following software tools were used in the experiments:

| Tools | Version | Usage | Source |
|---|---|---|---|
| pin_hic | V1.0.0 | Hi-C scaffolder | https://github.com/dfguan/pins |
| SALSA2 | V2.2 | Hi-C scaffolder | https://github.com/marbl/SALSA |
| BUSCO | V3.1.0 | Assembly assessment tool | https://gitlab.com/ezlab/busco |
| JupiterPlot | - | Circos plot tool | https://github.com/JustinChu/JupiterPlot |
| Dotplot | - | Dotplot tool | https://github.com/dnanexus/dot |

# 2 Scaffolding commands

### 2.0.1 Pin_hic command lines

Given raw Hi-C reads *hic(s)*, a draft assembly *asm* and iteration times *N*, we use the following commands to generate pin_hic scaffolds:

```
$ bwa index $asm
$ bwa mem -SP -B10 -t12 $asm $hic(s) | samtools view -b - > $bam(s)
$ samtools faidx $asm
$ pin_hic_it -i $N  -r $asm -x $asm.fai $bam(s)
```

### 2.0.2 SALSA2 command lines

Given a alignment bed file *bed* from Arima pipeline, a draft assembly *asm* and output directory *outdir* we use the following commands to generate SALSA2 scaffolds

```
$ python run_pipeline.py -a $asm -l $asm.fai -e GATC,GANTC -b $bed -m no -p yes -o $outdir
```

### 2.0.3 3D-DNA command lines

Given a mnd file *bed* from juicer pipeline, a draft assembly *asm* and output directory *outdir* we use the following commands to generate 3D-DNA scaffolds

```
$ bash 3d-dna-201008/run-asm-pipeline.sh -i 1000 -s 0 $asm $mndf
```

# 3 Supplementary tables and figures

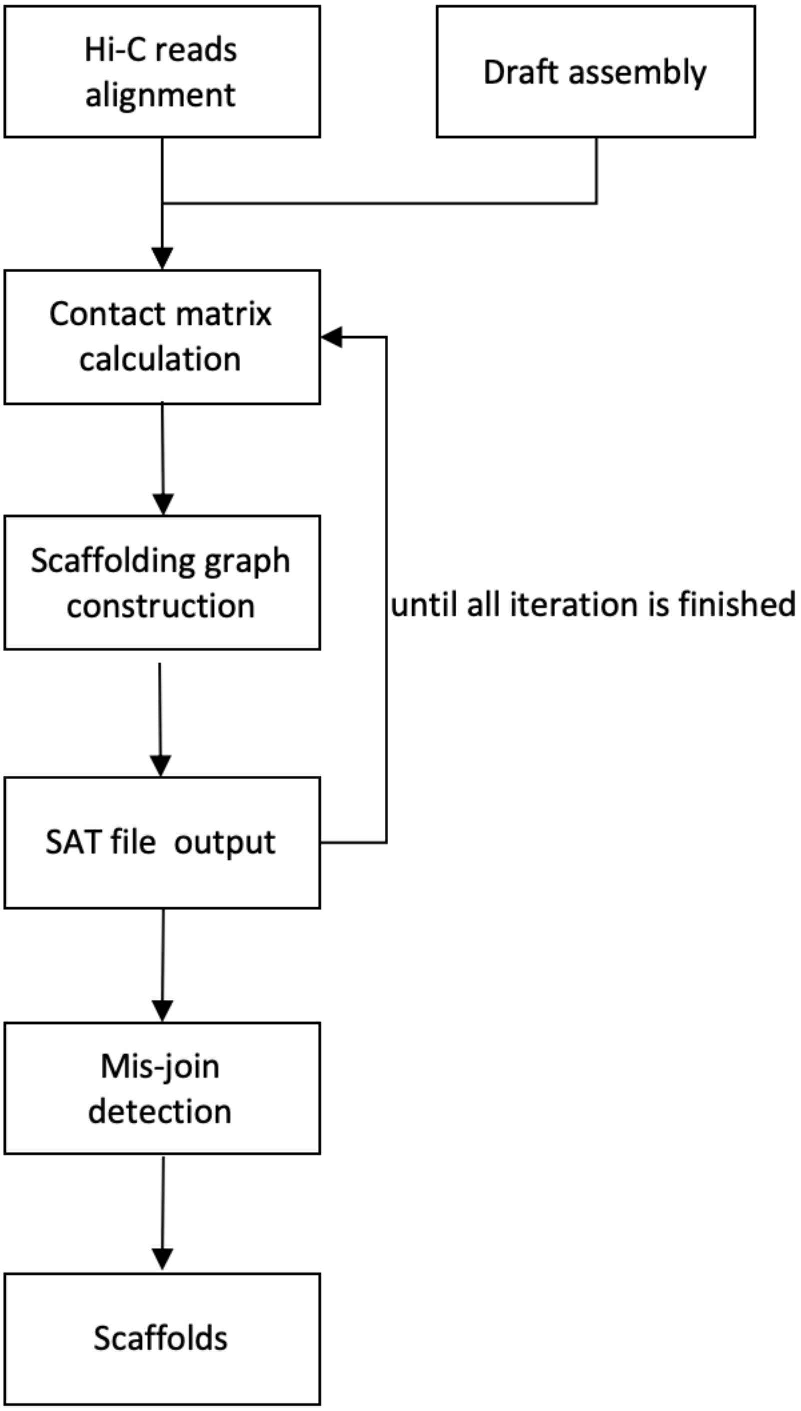


Supplementary Figure 1: Scaffolding diagram

Supplementary Table 1: SAT format

| | | |
|---|---|---|
| **H** | Header | |
| Field | Regular expression | Description |
| 1 | H | Label |
| 2 | VN:Z:[0-9]\.[0-9] | Version |
| **S** | Contig | |
| Field | Regular expression | Description |
| 1 | S | Label |
| 2 | .+ | Contig ID |
| 3 | [0-9]+ | Contig length |
| 4 | *[A-Za-z]+ | Contig sequence |
| **L** | Edge | |
| Field | Regular expression | Description |
| 1 | L | Label |
| 2 | .+ | Source contig ID |
| 3 | [-+] | Source orientation |
| 4 | .+ | Target contig ID |
| 5 | [-+] | Target orientation |
| 6 | wt:f:[0-9]*\.?[0-9]+ | Edge weight |
| **P** | Scaffold | |
| Field | Regular expression | Description |
| 1 | P | Label |
| 2 | [cu][0-9]{9} | Scaffold ID |
| 3 | [0-9]+ | Scaffold length |
| 4 | (.+[-+],)*(.+[-+]) | Ordered contig IDs |
| **A** | Scaffold set | |
| Field | Regular expression | Description |
| 1 | A | Label |
| 2 | a[0-9]{5} | Scaffold set ID |
| 3 | ([cu][0-9]{9},)*[cu][0-9]{9} | Scaffold IDs |
| **C** | Current scaffold set | |
| Field | Regular expression | Description |
| 1 | C | Label |
| 2 | a[0-9]{5} | Current scaffold set ID |

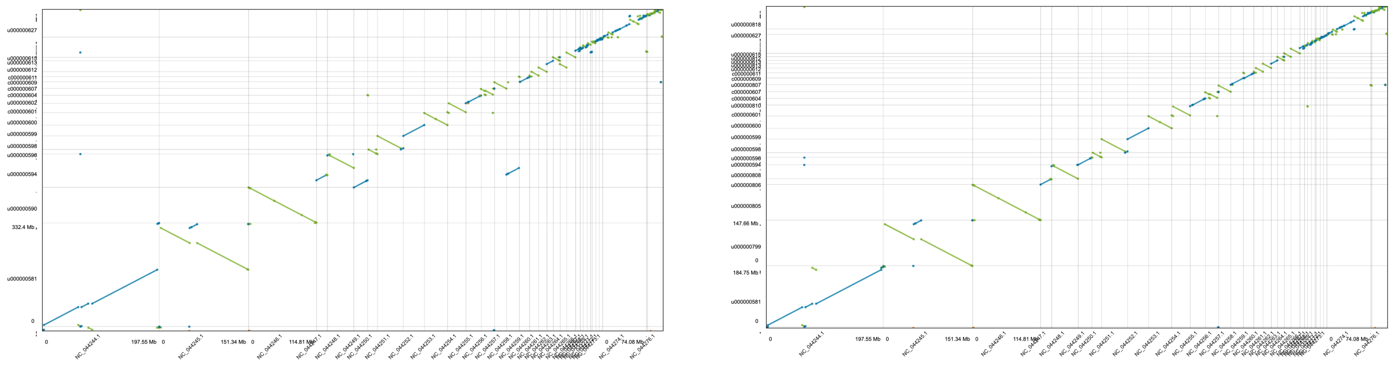

(a) (b)

Supplementary Figure 2: **Dotplot of pin_hic Cas scaffolds before and after misjoin detection** (**a**) pin_hic scaffolds alignments before misjoin detection and (**b**) pin_hic scaffolds alignments after misjoin detection. Both scaffolds were aligned to the VGP bCalAnn1 assembly. The horizontal axis represents the chromosomes in the reference genome and the vertical axis shows the scaffolds in pin_hic scaffolds. Before misjoin detection, the scaffold "u000000581" contains three inter-chromosome translocations, after the process, all the misjoins in the scaffold are removed, the scaffolds is aligned to the reference genome.

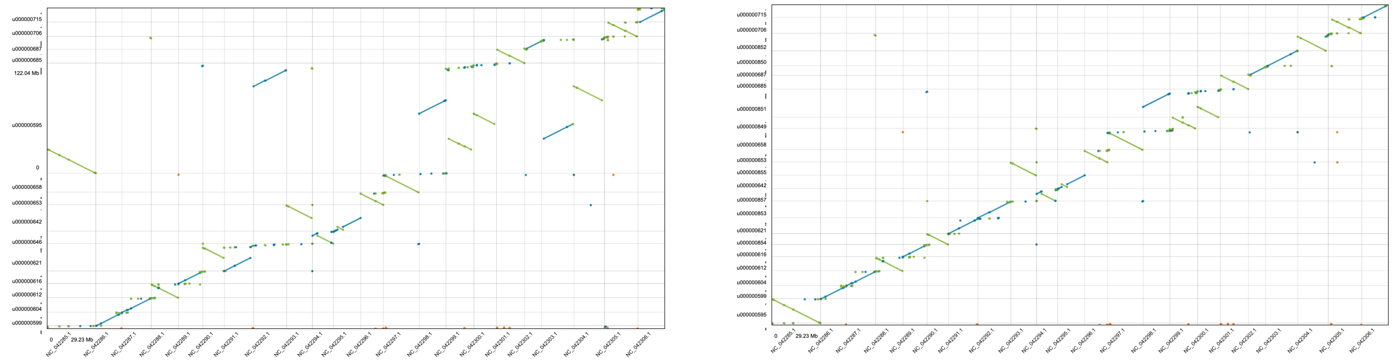

(a) (b)

Supplementary Figure 3: **Dotplot of pin_hic Trs scaffolds before and after misjoin detection** (**a**) pin_hic scaffolds alignments before misjoin detection and (**b**) pin_hic scaffolds alignments after misjoin detection. Both scaffolds were aligned to the VGP fTakRub1.2 genome. The scaffolds contain numerous misjoins before correction, although a few still remains after correction, the majority of them are removed.

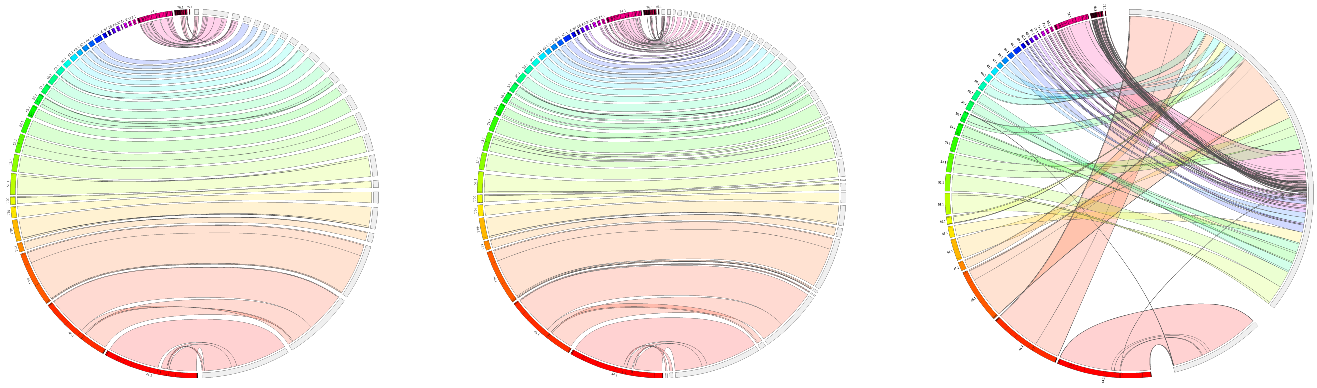

(a) (b) (c)

Supplementary Figure 4: **Scaffolds consistency plots of Cas scaffolds** (**a**) pin_hic scaffolds, (**b**) SALSA2 scaffolds and (**c**) 3D-DNA scaffolds. The largest 24, 43 and 2 scaffolds from pin_hic, SALSA2 and 3D-DNA scaffolds, consisting of 90% (N90) of the genome are aligned to the VGP bCalAnn1 chromosomes. Connections show aligned regions over 10 kb between the reference genome and the scaffolds. Large-scale mis-assemblies are visible as interrupting ribbons.

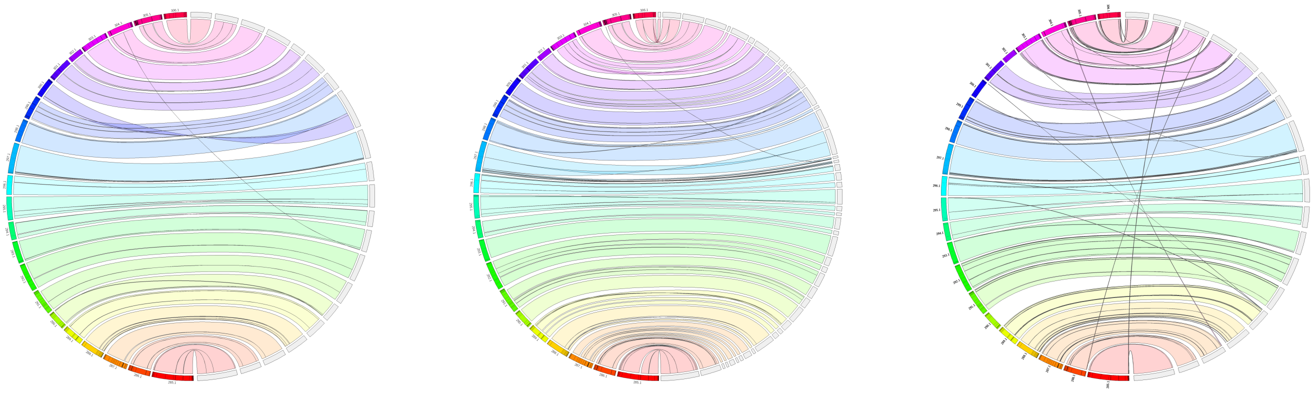

(a) (b) (c)

Supplementary Figure 5: **Scaffolds consistency plots of Trs scaffolds** (**a**) pin_hic scaffolds, (**b**) SALSA2 scaffolds and (**c**) 3D-DNA scaffolds. The largest 22, 56 and 19 scaffolds from pin_hic, SALSA2 and 3D-DNA scaffolds, consisting of 90% (N90) of the genome are aligned to the VGP fTakRub1.2 chromosomes. Connections show aligned regions over 10 kb between the reference genome and the scaffolds. Large-scale mis-assemblies are visible as interrupting ribbons.

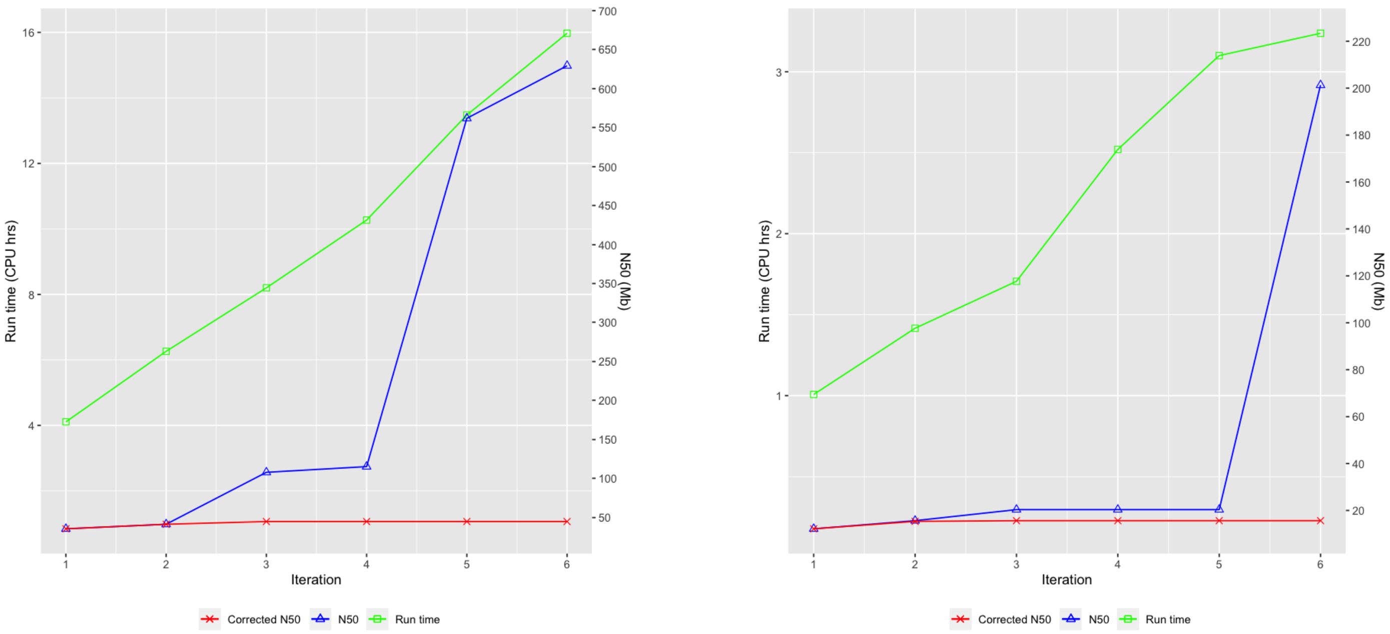


(a) (b)

Supplementary Figure 6: **Pin_hic run time and N50 variations in multiple iterations** (**a**) Cas scaffolds. (**b**) Trs scaffolds. The run time (dark green line) increases linearly with the iteration times, while the N50s grows rapidly at the $4^{\text{th}}$ round for Trs scaffolds, $5^{\text{th}}$ round for Trs scaffolds, the scaffold N50 is extended to 680 Mb for Cas and 235 Mb for Trs, and the corrected N50s reach 44.74 Mb for Cas and 15.75 Mb for Trs after the third round and remains stable after that.
